# COVID-19 and the Syndemic of Intimate Partner Violence, Mental Health, Substance Use, and HIV Care Engagement Among Black Sexual Minority Men Living with HIV in the US South

**DOI:** 10.3390/ijerph22071065

**Published:** 2025-07-03

**Authors:** Carrie L. Nacht, Hannah E. Reynolds, Chadwick K. Campbell, Kirstin Kielhold, Wilson Vincent, Daniel E. Siconolfi, Susan M. Kegeles, Erik D. Storholm

**Affiliations:** 1 School of Public Health, San Diego State University, San Diego, CA 92182, USA; hreynolds@sdsu.edu (H.E.R.); kkielhold@ucsd.edu (K.K.); estorholm@sdsu.edu (E.D.S.); 2 Herbert Wertheim School of Public Health and Human Longevity Science, University of California San Diego, La Jolla, CA 92093, USA; ckc003@health.ucsd.edu; 3 Department of Psychology and Neuroscience, Temple University, Philadelphia, PA 19122, USA; wilson.vincent@temple.edu; 4 RAND Corporation, Santa Monica, CA 90401, USA; dsiconol@rand.org; 5 Division of Prevention Science, Department of Medicine, University of California San Francisco, San Francisco, CA 94158, USA; susan.kegeles@ucsf.edu

**Keywords:** sexual minority men, COVID-19, syndemics, HIV care engagement

## Abstract

Background: Black sexual minority men (BSMM) are disproportionately affected by HIV incidence and have lower rates of HIV care engagement (e.g., retention in care, viral suppression), particularly in the US South. The COVID-19 pandemic exacerbated co-occurring psychosocial syndemic factors (e.g., depression, violence, substance use) that disproportionately impact BSMM living with HIV (BSMM+), but the impact of the pandemic on HIV care engagement among BSMM+ in the US South has not been explored in depth. Methods: BSMM+ (n = 27) were recruited from a longitudinal cohort in Dallas and Houston, Texas. Participants with less-than-optimal HIV care engagement, previous experiences of intimate partner violence (IPV), and prevalence of self-reported substance use were purposively selected for in-depth interviews from June 2022 to August 2023. Interviews lasted 54 min on average and were audio-recorded, transcribed, and de-identified before applying a thematic analysis approach. Results: Over half of participants experienced a relationship with IPV, used illicit substances in the past 60 days, and were depressed. Interviews highlighted that, during the COVID-19 pandemic, heightened levels of depression, substance use, and IPV individually and synergistically inhibited ART adherence and HIV care engagement. Participants described how IPV served to worsen their mental health and substance use, which, in turn, were the core drivers of poor HIV care engagement. Conclusions: The findings suggest that the COVID-19 pandemic exacerbated the syndemics of IPV, mental health, and substance use, and these acted as barriers to HIV care engagement. Future adherence interventions should synergistically address syndemic factors to maximize effectiveness.

## 1. Introduction

Black sexual minority men (BSMM) account for 25% of new HIV infections in the United States [1]. Over half of the nation’s incident HIV cases occur in the US South, and 60% of those diagnoses are among BSMM [2]. For people living with HIV (PLWH), retention and adherence to an HIV care regimen are crucial for maintaining viral suppression, which makes HIV non-transmissible [3]. Unfortunately, prior research has shown that racial disparities persist along the HIV care continuum, which consists of linkage to HIV care, retention in care, and achieving and maintaining viral suppression through regular adherence to antiretroviral therapy (ART). When diagnosed, BSMM living with HIV (BSMM+) have lower engagement in the HIV care continuum compared to sexual minority men (SMM) of other races and ethnicities [4,5,6]. One study in Georgia found that viral suppression was significantly less prevalent among BSMM than white SMM (67% and 79%, respectively) [4].

According to Syndemic Theory, lower HIV care engagement results from encountering numerous structural, psychosocial, and behavioral barriers manifested by the co-occurring epidemics of intimate partner violence (IPV), substance use, mental illness, violence, and HIV in sexual minority communities [7]. Studies have shown that a greater number of syndemic factors have been associated with decreased ART adherence and higher viral load among BSMM+ [8,9,10]. Intimate partner violence is a known risk factor for poor HIV care engagement and is commonly experienced by SMM [11,12,13,14], with an estimated 25–40% of all SMM experiencing IPV during their lifetime [15]. Intimate partner violence is associated with increased substance use among SMM [16,17,18], which has been associated with suboptimal adherence to ART [19,20,21,22]. BSMM who report IPV are also disproportionately impacted by mental health outcomes, such as depression [18]. Previous studies among BSMM+ showed that those with depressive symptoms had higher odds of suboptimal adherence to their ART regimen [23].

Since the COVID-19 pandemic, there has been a disproportionate increase in these syndemic risk factors across different racial and sexual minority groups. Previous research has shown that Black Americans in the rural South experienced worse mental health outcomes as a result of the pandemic [24]. Traumatic race-based violence events in 2020 and 2021, such as the murder of George Floyd and killing of Breonna Taylor, likely contributed to a steeper decline in mental health among Black Americans compared to white Americans during the pandemic [25,26]. Several studies have shown that alcohol use and other substances increased nationwide during the COVID-19 pandemic [27,28], but one study highlighted that Black adults reported initiation or increased substance use during the pandemic more than almost any other racial/ethnic group [29]. Furthermore, the incidence and severity of IPV were elevated during the COVID-19 pandemic compared to the three years prior [30,31,32]; IPV increased specifically among SMM as a result of social isolation regulations [32].

With these increased syndemic factors, it is not surprising that other studies have shown interruptions in HIV care engagement among SMM, particularly those belonging to racial and ethnic minority groups [33], and people living in the South [34,35]. However, previous studies assessing the impact of COVID-19 on HIV care engagement have largely focused on outcomes (e.g., viral suppression) and not assessed the psychosocial syndemics (e.g., substance use, mental health, IPV) that impact those outcomes [36,37,38]. As such, the objective of this study is to explore how the COVID-19 pandemic and associated public health measures exacerbated psychosocial syndemics that affected HIV care engagement for BSMM+ in the South. These results may be used to inform clinicians, public health officials, and PLWH how to maintain adherence in a HIV care regimen during future times of social and economic disruptions or crises.

## 2. Materials and Methods

### 2.1. Study Design and Recruitment

The participants of this study were recruited from a larger, community-based longitudinal cohort of BSMM living with HIV in the US South. The participants in the parent cohort were recruited using long-chain referral methods [39] in Dallas and Houston, TX, in 2015–2016. Participants engaged in four waves of survey data collection spanning from 2015 to 2022. Detailed recruitment methods have been described elsewhere [40]. Briefly, baseline participants were recruited using long-chain peer referral methods based on respondent-driven sampling [39,41,42]. These participants were identified as well-liked and popular in the BSMM community, who were then asked to recruit other BSMM in their social networks. A smaller proportion of participants were recruited through a variety of methods (e.g., flyers, social media). Participants were eligible if they identified as male, Black or African American, were 18–29 years old at the time of enrollment into the cohort (in 2015–2016), lived in the metropolitan area of Houston or Dallas, and were found to be living with HIV when they underwent HIV testing prior to the baseline assessment.

### 2.2. Data Collection

For this qualitative sub-study, cohort participants were contacted via email and were invited to participate in a one-time semi-structured interview regarding their experience with HIV care during the COVID-19 epidemic. Participants were purposively sampled for this analysis to include (1) men who reported less than optimal HIV care engagement in the past year (defined as missing ART doses in the past year, detectable or unknown viral load, or not currently on ART) or (2) those who reported current or previous experiences of IPV and/or high levels of substance use [43,44,45]. Only a subsample of participants who met these criteria were invited to participate in this optional supplemental interview. One of the study’s investigators (CKC), a self-identified Black gay man from the Deep South, conducted all interviews (n = 27) in English via Zoom^®^ from June 2022 to August 2023. The investigators stopped collecting data after 27 interviews when interviews were determined to have reached “information power,” or when the researchers determined that they had collected enough data to sufficiently analyze the data to answer the research question [46]. The semi-structured interviews lasted 30–70 min (average 54 min). The interview guide included questions on the effects of the COVID-19 pandemic on HIV diagnosis and living with HIV, barriers and facilitators to HIV care, HIV stigma, and IPV (e.g., How were your sexual and romantic relationships with other people been affected by the COVID-19 pandemic? Have you noticed an increase in anger or frustration with each other because of issues related to COVID? Tell me about how the pandemic has affected your relationship with your HIV doctor/provider? Generally speaking, what has the pandemic been like for you?).

All study procedures were reviewed and approved by the Institutional Review Board at San Diego State University. Prior to the interview, participants were sent a copy of the consent form. At the time of the interview, the facilitator reviewed the consent form and answered questions. Each participant provided verbal consent, which was obtained to protect participant privacy. No qualitative study data were directly associated with a participant’s identifying information. We used pseudonyms in the reporting of qualitative findings. Participants received a USD 75 gift card for participating in the study after the interview was completed.

### 2.3. Data Analysis

A team of three qualitative analysts (CKC, HER, KK) coded all the interviews using a codebook thematic analysis approach [47,48,49]. Audio-recorded interviews were professionally transcribed verbatim by an independent transcription agency. The three analysts read all interview transcripts to become familiar with the data and check for transcription accuracy, while taking notes of potential themes and codes by memoing. Initially, each analyst independently open-coded one transcript, then discussed and merged similar codes, eliminated redundant codes, and reconciled discrepancies. This process was repeated with two additional transcripts before agreement on the meaning and application of codes was reached. Decision trails were kept to ensure rigor and consistency. Once the codebook was established, team members independently coded all transcripts. The analysts continued to meet biweekly to modify the codebook as they continued to code all the interviews [50,51]. The themes for this analysis were identified by extracting code reports related to COVID-19 and HIV, mental health, substance use, IPV, and social support. All qualitative data analyses were performed using Dedoose (v9.0.17, Los Angeles, CA, USA) [52].

## 3. Results

### 3.1. Participant Characteristics

A total of 27 BSMM living with HIV participated in semi-structured interviews. Participants were on average 30.81 years old. About half of the participants had at least some college education (52%) and were employed full-time (44%). Most self-identified as gay (78%) and had either private or public health insurance (70%). All but two participants self-identified as a man, with two participants additionally identifying as transgender or non-binary (7%). More than half (63.0%) of the participants reported having been in a relationship with IPV, having used an illicit substance in the past 60 days (55.6%), and were clinically depressed (51.9%) (Table 1).

Interviews took place on average 183 days (range: 35–386 days) after participants completed their most recent quantitative study survey, which happened between May and October of 2022, and had been living with HIV an average of 10.85 years (range: 6–17 years). At the time of the survey, most (85.2%) reported that they were currently on ART and were undetectable at last viral load test (77.8%), although there was a highly varied reported number of missed ART doses in the past 60 days, ranging from no days (22.2%) to 4–7 days a week (14.8%). Six participants (22.2%) reported missing ART for seven or more days in the past sixty days.

### 3.2. Thematic Analysis

In interviews about the impact of COVID-19 on HIV care engagement, three key themes were identified from these data surrounding the impact of syndemic conditions on HIV healthcare engagement during the COVID-19 pandemic: 1) substance use as a response to COVID-19-related strains, 2) COVID-19 as an additional stressor on mental health, and 3) a confluence of COVID-19 and psychosocial syndemics. Each quote is followed by the participant’s pseudonym and their age at the time of the interview. The themes and respective illustrative quotes can be found below.

Substance Use as a Response to COVID-19-Related Strains

Participants described a variety of ways substance use affected their care during COVID-19, including self-described ‘forgetfulness’ and ‘carelessness’, or a conscious effort to avoid drug–drug interaction between HIV medication and recreational substances. Participants disclosed that elevated substance use during the pandemic, including methamphetamine, cocaine, and alcohol, impeded their ability to take their HIV medication as prescribed. Paul started drinking heavily in response to stress and uncertainty related to the COVID-19 pandemic:

“The drinking, that’s picked up extremely a lot… I probably wasn’t taking my medicine at all. I was probably too drunk to take it.” (Paul, 26). Like Paul, other men described how boredom and isolation during the pandemic led to more substance use. Mason described how the pandemic “just made me want to just drink and get high more because I was bored and stuff like that.” (Mason, 31). Keith’s methamphetamine use increased during the pandemic and affected his ability to attend his appointments regularly and adhere to his medication.

I was smoking already before COVID. But like when COVID hit, I started like hardcore binging. I would be gone for weeks. I’ll be gone for two weeks out of the month like, just smoking, just smoking and smoking… During the pandemic, it was very rare [that I saw my doctor]. Very rare. I would have appointments, and I would go to my appointments, and then they’ll have a follow-up appointment, and I’ll miss it. Why? Because I’d be binging [meth].

(Keith, 30)

These men illustrate the ways in which COVID-19, an acute societal stressor that leads to economic and social uncertainty, can interact with and exacerbate pre-existing psychosocial syndemic conditions. That interaction influenced HIV care engagement creating additional health impacts beyond the COVID-19 virus itself.

Other participants highlighted that they made a conscious decision to avoid mixing their ART medication with other substances. For example, Jamar described skipping ART doses when he was using cocaine.

When the pandemic started, and we went on lockdown that was about the time when I was dating this person. I noticed that they had an addiction to cocaine, so obviously being around this person I indulged in it as well… I wasn’t really into any of that, but I guess because I was just around them so much that I just started to do it. So, that definitely did have a factor because this is a global pandemic and I’m just at home so much it didn’t help… [it affected my ability to] take my medication because I’d be like, ‘well, I shouldn’t take it right now because I’m doing [cocaine]… I’m like, ‘well, if I’m taking ibuprofen, I wouldn’t want to drink’ or ‘I’m taking any other type of medication, I wouldn’t want to drink or do anything else or do any type of drugs. So, I probably shouldn’t take [my HIV medication] if I’m doing any drugs.’

(Jamar, 26)

Similarly, when Chris was asked about whether meth use impacted his ability to take his HIV medications, he responded affirmatively: “Probably so. Yes, as in forgetfulness. Or laziness, or, ‘Oh, I have to go get [meth] out of my system because I’m high [before I] take the medicine.’” (Chris, 33). Increased substance use was a common response to the stress of a global pandemic that had direct effects on their ability to manage their HIV care.

COVID-19 as an Additional Stressor on Mental Health

While descriptions of mental health symptoms varied among participants, most participants who experienced a mental health decline during the COVID-19 pandemic stated that their declining mental state negatively impacted their ability to adhere to their ART medications or attend appointments. For example, when asked how the pandemic affected his life, Sam described, “I was very much depressed. Very much depressed. I didn’t tell anyone, but I was literally depressed… I forget [to take my HIV medication] half the time.” (Sam, 34).

Importantly, for some men, COVID-19 occurred in concert with other stressors in their lives. In the preceding section, we described how Paul’s drinking increased during the pandemic. He described how this was in response to his being laid off at the beginning of the pandemic and the general uncertainty of that time. In addition, his fiancé died less than a year prior to our interview. When prompted about how this trauma affected his experience with his medication regimen, Paul explained that his loss of interest in life led to inconsistency with his ART regimen.

I didn’t really care about anything. Didn’t really care about life, or anything… Woke up, went to sleep… I have my days where I don’t [take my medication]… but I’m going to get better as far as taking it every day. I just got to get out of this mood. I have my moods. I have my moments that’s where, you know, sometimes I wake up with the world on my shoulders.

(Paul, 26)

Similar to Paul, Jesse also experienced periods of depression during the pandemic that led him not wanting to care for himself.

[The pandemic] put me in a state of mind. To be honest, it put me in a depressing state of mind, and sometimes I got to the point to where I didn’t care if I took my medicine or not. I didn’t care if I was dead or alive. I lost focus. I mean, it sounds bad, but it’s just the honest truth. Basically, I was getting to the point where I was feeling like I was giving up, basically. (Jesse, 28)

The COVID-19 pandemic created circumstances that are known to have negative effects on mental health. Jesse described that, during the pandemic, he and his partner struggled with job loss, financial strain, and conflict within their relationship, all of which led to his depression.

Darius described how his pre-existing depression was worsened because he gained weight when taking his ART medications—a common side effect. This worsening depression, unstable housing, inability to maintain a job, and a lack of transportation contributed to him not taking his medication.

When I started taking that medicine, it made me feel like really heavy, and messed with my appetite, and made me feel like I was really hungry. Knowing that I have depression and major stress on me, it doesn’t help. […] During Corona, I probably went to the doctor twice in that whole year. You see what I’m saying? I took the medicine that they gave me. I did have some backup Triumeq in store that I have left over, and I used that to make up for the times I could not go to the doctor. Like no buses, or everything was just really packed, and it really put me at a disadvantage. I did collect some major depression at that time, when I was not able to take care of myself.

(Darius, 32)

Importantly, he also noted that his declining mental health led to an increased use of substances as a way of coping. Due to his worsening situation, he sought out substances.

When that depression kicks in, like nothing is ever going to matter or help, that’s when drugs become a comfort unit for people. If I can’t get into my doctor, and I can’t get this job… Do you see how all that piles on the top of each other that makes a person want to go and say, ‘Hey, I’m just going to go get high’?

(Darius, 32)

Travis contracted COVID-19 and had to be hospitalized, leading him to fall into depression. As a person living with HIV, having COVID-19 caused him considerable fear and anxiety.

Oh my god, this HIV with [COVID-19] is going to take me out. I thought I was going to die, I really thought I was going to freaking die, I was counting my days… [COVID] literally made me feel like I was going to die just because of the fact HIV and another strong sickness worldwide that they’re saying is killing people. So I did fall into a depression stage. I was depressed as hell. I was like, I feel like this season I might die… Even after I was cured of [COVID], I was still depressed because I was scared.

(Travis, 26)

Uniquely, Travis diverged from the rest of the participants by explaining how this near-death experience made him continue seeing his doctor for HIV treatment and take his daily ART due to fear of the potential resulting consequences. “I’m going to take [my daily ART medications]. I’ve taken it every day just because I’m scared to not take it.” (Travis, 26).

Overall, while poor mental health manifested differently, these data show that living through the pandemic and its associated societal impacts affected participants’ mental health and, for some, their ability to adhere to HIV care.

A Confluence of COVID and Psychosocial Syndemics

Participants described how experiencing the pandemic exacerbated physical violence in their relationship and, for some, led to IPV for the first time in relationships with no history of violence. When prompted about how COVID-19 impacted his relationship, Aaron responded that, after the pandemic started,

We argued all the time about little stupid stuff… during that time it allowed us to see things that we liked and we did not like about each other… Plenty of physical altercations. I will say that I did start a fair few of them in the beginning before 2021. But after I graduated and it hit 2022, I stopped doing a lot of those things. I stopped being the antagonizer.

(Aaron, 26)

He also detailed how his volatile relationship, coupled with substance use, impacted his mental and physical health.

If me and him were arguing, I wouldn’t even want to eat because my appetite was gone… it got to the point where I needed to see a therapist… [Our relationship] was so unhealthy. And I feel like I was literally watching myself wither away.

(Aaron, 26)

Likewise, Gerald discussed how the abuse in his relationship impacted his mental health. He explains that his partner was unfaithful, which made their sexual health less safe. “[The abuse] affected my mental health more than my physical health… there was infidelity and stuff… we could have been safer.” (Gerald, 32).

In the previous section, we described the toll that COVID-19-related strains took on Jesse’s mental health and his ability to take his HIV medications. One factor contributing to his poor mental health was IPV. He described having a good relationship with his partner until sheltering in place led to violence.

Last year, 2021, I was stabbed bad. By my old partner, so, it’s just, yeah, it got very hectic. […] I had to go to a mental institution. It really messed me up… I was in the hospital for months, from July to like, October, the beginning of November.

(Jesse, 28)

Jesse further described how his experience with IPV and the subsequent institutionalization had a negative impact on his HIV regimen: “I’d say the most that I ever went [without HIV medication], probably about seven or eight months.” (Jesse, 28).

Jamal highlights how during the pandemic, intimate partner violence and mental health became very intertwined, and that he used substances as a way to cope:

“Two addicts cannot be together… violence is typically something that is present… I definitely think [abuse] affected my health because… my self-esteem had been affected through that relationship. At the same time you factor in seeking healthcare options and no one point you [to any]… and then, hey, here’s somebody with a solution, that [meth] makes you perfectly fine, you forget all of your problems. And it takes you down a completely different pathway.

In this case, participant narratives illustrate how the COVID-19 pandemic, substance use, IPV, and mental health converged in the lives of BSMM+ in the South. Importantly, these data provide novel insight into how periods of acute social stress can exacerbate pre-existing psychosocial syndemics and amplify their negative effects on the ability of BSMM+ to take care of their health.

## 4. Discussion

This study is one of the first to examine how the COVID-19 pandemic and the associated public health measures exacerbated psychosocial syndemics that affected HIV care engagement for BSMM+ in the South. The participant narratives in our study illustrate how the COVID-19 pandemic increased substance use and IPV, negatively impacted mental health, and led to disruptions in HIV care engagement as a result. Previous research has already established that SMM experiencing a multitude of psychosocial syndemics, such as depression, anxiety, alcohol use, and substance use, has been associated with lower engagement in the HIV care continuum—namely, medication non-adherence and viral suppression [54,55]. The COVID-19 pandemic was a period of acute and intense social and economic stress that had a disproportionate, negative impact on communities of color, sexual and gender minority communities, and other marginalized communities [56,57,58,59]. Because of COVID-19’s impact on vulnerable populations, COVID-19 may be thought of as an additional *syndemic* rather than a *pandemic*, given its interaction with socially stigmatized populations living in economic precarity [60]. As such, maintaining engagement in healthcare—particularly HIV care, which already faces a multitude of barriers—is more difficult in states of emergency. Furthermore, studies have shown that HIV-related stigma contributes to worse care outcomes, such as suboptimal medication adherence and lower retention in HIV care among PLWH [61,62,63,64]. These phenomena are exacerbated among BSMM living with HIV due to the racial stigma and discrimination they encounter, in addition to sexuality and HIV-status discrimination; the negative effects of multiple psychosocial syndemics on HIV care outcomes—namely, medication non-adherence—among SMM of color are well documented [10,65,66].

This study builds upon the existing literature surrounding syndemic theory by showing that, during times of social and economic disruption such as the COVID-19 pandemic, certain syndemic conditions are exacerbated, resulting in changes in HIV care engagement among BSMM+ in the US South. Programs addressing intersecting syndemics in HIV care engagement must address the critical role of structural racism and discrimination, especially during unprecedented times. These programs should actively implement anti-racist policies, provide cultural competency training, diversify the healthcare workforce, and advocate for systemic changes to reduce health disparities and improve access to care for marginalized populations.

There are limitations to this study. First, this analysis was made up exclusively of BSMM living with HIV from two large, urban cities in the US South. Although this work provides valuable insight into this disproportionately impacted population, caution should be taken in generalizing these findings to other BSMM. Second, interviews were conducted an average of six months after participants completed the quantitative surveys, from which the qualitative interviews were sampled. Thus, some participants may not have been experiencing COVID-19 stressors or the related syndemic IPV, substance use, and mental health burdens they reported during their survey. As such, participants were asked to reflect on their experiences during COVID-19. Finally, all qualitative research is subject to self-selection bias, wherein participants who agreed to be interviewed may not be representative of the broader population.

Despite these limitations, this analysis provides valuable knowledge about the influence of the COVID-19 syndemic on factors related to engagement in the HIV care continuum among BSMM living with HIV living in the South. It also points to the critical role of social support to mitigate barriers to care engagement during a time of social and economic stress. Given limited research on the impact of the COVID-19 pandemic on HIV care for this population in the South, this study provides important recommendations for interventions needed to address the social barriers to sustained engagement in care with the goal of improving clinical outcomes and the overall health and well-being of BSMM living with HIV in the US South.

This article is a revised and expanded version of a paper entitled “Syndemic intimate partner violence, mental health, substance use, and ART non-adherence among Black Sexual Minority Men Living with HIV in the US South,” which was presented at the International Association of Providers of AIDS Care (IAPAC)’s Fast-Track Cities 2023 conference, 25–27 September 2023, Amsterdam [64].

## Figures and Tables

**Table 1:** Participant characteristics (n = 27).

Demographics	
Age, mean (SD)	30.8 (2.9)
Highest level of education	n (%)
High school graduate/GED	7 (25.9)
Some college, AA or technical degree	14 (51.9)
Bachelor’s degree or any graduate studies	6 (22.2)
Sexual orientation	
Gay	21 (77.8)
Bisexual	6 (22.2)
Gender identity *	
Man	27 (100)
Employment	
Full Time	12 (44.4)
Part-Time	4 (14.8)
Unemployed	11 (40.7)
Annual Income	
less than USD 10,000	6 (22.2)
USD 10,000–39,999	10 (37.0)
USD 40,000–79,999	8 (29.6)
Health insurance	
Yes	19 (70.4)
No or unknown	8 (29.7)
**HIV care characteristics**	
Years since diagnosis, median (range)	10 (6–17)
Currently on ART	23 (85.2)
On ART for the past 60 days	22 (81.5)
HIV viral load at last test	
Undetectable	21 (77.8)
Detectable	5 (18.5)
Don’t know	1 (3.7)
Days missed ART dose in past 60 days	
Never	6 (22.2)
Once a week or less than once a week	6 (22.2)
2–3 days a week	6 (22.2)
4–7 days a week	4 (14.8)
Missed ART for 7+ consecutive days in past 60 days	6 (22.2)
**Syndemic characteristics**	
Ever experienced a relationship with IPV	17 (63.0)
Illicit substance use in the past 60 days, not including cannabis	15 (55.6)
Depression **	
Not depressed	8 (29.6)
Distressed	3 (11.1)
Depressed	14 (51.9)

* Not mutually exclusive. ** As measured by the Center for Epidemiological Studies-Depression scale, using clinical categories [53].

## Data Availability

The datasets presented in this article are not readily available because the data are part of an ongoing study. Requests to access the datasets should be directed to Chadwick Campbell, ckc003@health.ucsd.edu.
